# A systematic review on using intervention mapping to guide behavioral interventions for improving stroke patient outcomes

**DOI:** 10.3389/fmed.2026.1850511

**Published:** 2026-06-29

**Authors:** Jinxian Yang, Jianing Liu, Xiaoxia Zeng, Fan Yang, Hui Zhang, Yongxia Ding

**Affiliations:** 1 Shanxi Medical University, Taiyuan, China; 2 Department of Neurology, Ward 6, Shanxi Cardiovascular Hospital, Shanxi Provincial Key Laboratory of Precision Diagnosis and Treatment of Heart Failure, Cardiovascular Hospital Affiliated to Shanxi Medical University, Taiyuan, China; 3 Department of Nursing, Shanxi Cardiovascular Hospital, Shanxi Provincial Key Laboratory of Precision Diagnosis and Treatment of Heart Failure, Cardiovascular Hospital Affiliated to Shanxi Medical University, Taiyuan, China; 4 Department of Neurosurgery, Ward 1, Shanxi Cardiovascular Hospital, Shanxi Provincial Key Laboratory of Precision Diagnosis and Treatment of Heart Failure, Cardiovascular Hospital Affiliated to Shanxi Medical University, Taiyuan, China; 5 Department of Neurology, Ward 5, Shanxi Cardiovascular Hospital, Shanxi Provincial Key Laboratory of Precision Diagnosis and Treatment of Heart Failure, Cardiovascular Hospital Affiliated to Shanxi Medical University, Taiyuan, China

**Keywords:** behavioral interventions, intervention mapping, rehabilitation, stroke, systematic review

## Abstract

**Background:**

Functional recovery after stroke is substantially dependent on systematic, evidence-based behavioral interventions. Intervention Mapping (IM), as a structured framework for intervention development, has demonstrated favorable outcomes across various health domains. This systematic review had one primary aim to identify and characterize how the IM framework has been applied to develop behavioral interventions for stroke survivors. Moreover, we describe the core characteristics of IM-guided stroke behavioral interventions and evaluate the methodological reporting quality of IM application.

**Methods:**

A systematic search was conducted in PubMed, Embase, Cochrane Library, Web of Science, and CINAHL databases for relevant literature published up to March 20, 2026. Studies employing IM to guide the development of behavioral interventions for stroke survivors were included, irrespective of study design. The characteristics of intervention design, implementation processes, methodological quality, and outcome evaluations were synthesized and analyzed.

**Results:**

Nine studies were included, with overall methodological quality ratings ranging from “moderate” to “strong.” Three patterns of IM application were identified: (1) full application of all six steps (4/9); (2) partial application focusing on the development phase (Steps 1–4, 4/9); and (3) deep integration with co-design methodologies (2/9). All interventions integrated behavior change theories and exhibited multi-level, multi-component, and contextually tailored characteristics. Delivery formats included face-to-face counseling, telehealth coaching, digital toolkits, and video-based education. Six studies reported feasibility evaluations; interventions demonstrated high acceptability, appropriateness, and feasibility, with preliminary signals of improved knowledge, enhanced self-efficacy, and positive behavioral intentions. No studies reported long-term hard clinical outcomes. All studies produced logic models and intervention manuals; however, reporting transparency regarding operational details of Step 2 (matrices of change objectives) and Step 3 (theory-based methods and practical strategies) was suboptimal in some studies, constraining methodological replicability.

**Conclusion:**

IM provides a viable structured framework for the systematic development and implementation of behavioral interventions in stroke rehabilitation, contributing to enhanced theoretical grounding, contextual adaptability, and implementation rigor. Future research should deepen the internalization of IM’s technical core, rigorously adhere to reporting standards, and advance mature interventions into large-sample, long-term, multi-center efficacy trials to achieve systematic orchestration across the intervention science continuum—from development to clinical translation.

**Systematic review registration:**

The Systematic Review was registered in INPLASY platform (INPLASY202630004), the website was: https://inplasy.com/inplasy-2026-3-0004/.

## Introduction

Stroke, as one of the leading causes of disability and mortality worldwide, imposes a substantial burden on patients, families, and healthcare systems (1, 2). While acute treatment methods continue to advance, long-term functional recovery and quality-of-life improvement after stroke largely depend on sustained, standardized, and evidence-based behavioral interventions, such as rehabilitation exercise, self-management, and lifestyle adjustments (3). Beyond functional recovery, contemporary stroke rehabilitation goals emphasize successful reintegration of patients into family, work, and social roles, with patient satisfaction serving as a critical indicator of truly positive and minimally traumatic reintegration (4). However, behavioral intervention studies have historically prioritized clinical and functional endpoints over these psychosocial reintegration outcomes. Intervention strategies frequently lack systematic theoretical guidance and are insufficiently aligned with patients’ actual needs and cultural contexts; the development process of intervention protocols is often not transparent enough to allow replication or widespread adoption; moreover, evaluation of the implementation process and underlying mechanisms is often overlooked, limiting the optimization and dissemination of intervention outcomes (5).

In this context, adopting a systematic approach to plan, design, and evaluate behavioral interventions is particularly important. Intervention Mapping (IM) is a stepwise planning framework comprising six specific steps: (1) needs assessment, (2) specification of performance and change objectives organized into matrices, (3) selection of theory-based methods and practical strategies, (4) intervention production, (5) implementation planning, and (6) evaluation planning. Unlike traditional intervention development approaches that may rely on implicit assumptions or single theories, IM requires developers to explicitly document the logical chain from problem analysis to intervention components, thereby enhancing transparency, replicability, and theoretical grounding (6). This method has been successfully applied in various fields such as chronic disease management and public health promotion (7, 8). Its core strengths lie in emphasizing logical modeling, establishing multi-level change objectives, and integrating behavior change theories with empirical evidence and stakeholder input, thereby enhancing the scientific rigor, acceptability, and practical feasibility of interventions (9).

Despite the clear methodological advantages of IM, its application status, common experiences, and practical effectiveness in the field of behavioral interventions for stroke rehabilitation have not yet been systematically reviewed and summarized. There is currently a lack of comprehensive evaluation regarding the application patterns of this method in this specific population, the challenges encountered, and its potential contribution to intervention outcomes. Therefore, a systematic integration and analysis of existing relevant studies is warranted. This study aims to comprehensively review and analyze the existing evidence on the use of IM to guide the development of behavioral interventions for stroke patients through a systematic review. The “behavioral interventions” were defined as any structured program aiming to change stroke survivors’ actions or habits relevant to recovery and secondary prevention, including but not limited to: rehabilitation exercise adherence, self-management behaviors (e.g., medication taking, blood pressure monitoring), physical activity promotion, sedentary behavior reduction, dietary modification, and return-to-work behaviors. Interventions focusing exclusively on pharmacological, surgical, or device-based treatments without a behavioral component were excluded. It seeks to outline the development processes and core characteristics of such interventions and to evaluate the application methods and extent of IM in stroke behavioral interventions.

## Methods

### Search strategy and selection criteria

This study adhered to the Preferred Reporting Items for Systematic Reviews and Meta-Analyses (PRISMA) guidelines to conduct a systematic search, screening, data extraction, and synthesis of research on the application of IM in guiding behavioral interventions for stroke patients (10). The systematic review protocol was prospectively registered with the International Platform of Registered Systematic Review and Meta-analysis Protocols (INPLASY) on March 02, 2026 (registration number: INPLASY202630004). Literature screening commenced on March 20, 2026, following protocol finalization. No deviations from the registered protocol occurred during the conduct of the review.

A systematic search was performed in the following electronic databases: PubMed, Embase, the Cochrane Library (CENTRAL), Web of Science, and CINAHL. The search timeframe covered from database inception to March 20, 2026. The search strategy was constructed based on three core concepts: “stroke,” “Intervention Mapping,” and “behavioral intervention,” utilizing a combination of subject headings (e.g., MeSH) and free-text terms, with adjustments made according to the specific features of each database. The detailed search strategies for each database are provided in Supplementary File 1. Additionally, the reference lists of included studies were manually screened to identify potentially relevant publications.

The literature screening process was conducted independently by two researchers. Initial screening was performed by reviewing titles and abstracts, followed by a full-text assessment of potentially eligible articles. Any disagreements were resolved through discussion or consultation with a third researcher. Inclusion criteria were established according to the PICOS framework: (1) Participants: stroke patients (including ischemic and hemorrhagic stroke, at any disease stage), or their caregivers and rehabilitation team members. If a study population included patients with other conditions, data specifically related to stroke patients had to be extractable separately; (2) Interventions: behavioral interventions explicitly reported as being developed under the guidance of IM (employing complete or partial IM steps). Behavioral intervention content included, but was not limited to: promotion of rehabilitation exercise adherence, self-management, increasing physical activity, cognitive training, and lifestyle modification; (3) Comparisons: no restrictions were applied, allowing for comparisons with usual care, other active interventions, or study designs without a control group; (4) Outcomes: primary focus was placed on characteristics of the intervention development process, indicators of intervention feasibility/acceptability, and patient-level health or behavioral outcomes. To be classified as “using IM,” a study had to explicitly state that IM guided the intervention development process and provide sufficient methodological detail to identify which IM steps were applied. Studies were categorized as “full application” if they reported completing all six IM steps. Studies were categorized as “partial application” if they reported completing Steps 1–4 only, regardless of whether this was by design or due to incomplete reporting. Studies that integrated IM with co-design methodologies were coded separately but retained within the partial or full application category based on step coverage; and (5) Study design: all studies applying IM in intervention development were included, encompassing intervention development studies, feasibility/pilot studies, randomized controlled trials, and non-randomized controlled studies, among others. Literature that merely mentioned the IM concept without its practical application in the intervention development process, review articles, commentaries, conference abstracts, and studies for which full text was unavailable were excluded. Moreover, grey literature (e.g., conference proceedings, dissertations, institutional repositories) and trial registries (e.g., clinicalTrials.gov, WHO ICTRP) were not searched, which may introduce publication bias. Non-English databases were not searched.

### Data collection and quality assessment

Two investigators independently extracted information using a pre-designed data extraction form. Data items included: author(s), publication year, country, study objectives, study design, sample size, participant characteristics, details of IM application (specific IM steps followed [Steps 1–6], core activities and outputs for each step, integrated behavior change theories, mode and degree of stakeholder involvement), intervention content (intervention name, specific components, delivery format, intensity, duration, and implementers), and outcome measures (intervention development outputs, feasibility/acceptability assessments, and preliminary effectiveness evaluations). The methodological quality of included quantitative studies was assessed using the Effective Public Health Practice Project (EPHPP) Quality Assessment Tool (11). This tool systematically evaluates study quality across eight domains. Each domain is independently rated according to predefined criteria, culminating in an overall global quality rating for each study. A ‘strong’ global rating required no ‘weak’ ratings across any domain; a ‘moderate’ global rating permitted one ‘weak’ rating; and a ‘weak’ global rating required two or more ‘weak’ ratings. The “not applicable” ratings for domains that are conceptually irrelevant in studies not designed as comparative effectiveness trials. This appraisal was conducted independently by two investigators. Any discrepancies were resolved through discussion or, if necessary, arbitration by a third investigator.

### Data analysis and synthesis

Given the anticipated substantial heterogeneity among the included studies regarding intervention content, target populations, outcome measures, and research methods, this study will primarily employ a narrative synthesis approach. The findings will be structured and summarized according to the following thematic areas: (1) application patterns of IM: analyzing the frequency, depth, and variations in the application of specific IM steps; (2) characteristics of the intervention programs: summarizing core components, theoretical frameworks, and delivery modes; (3) development process and feasibility: integrating results related to stakeholder engagement, pilot testing, and feasibility assessments; and (4) intervention effectiveness: descriptively reporting the effects of interventions on patient behaviors, functional outcomes, and health-related outcomes, categorized by outcome domains.

## Results

### Literature search and study selection

**Figure 1:**
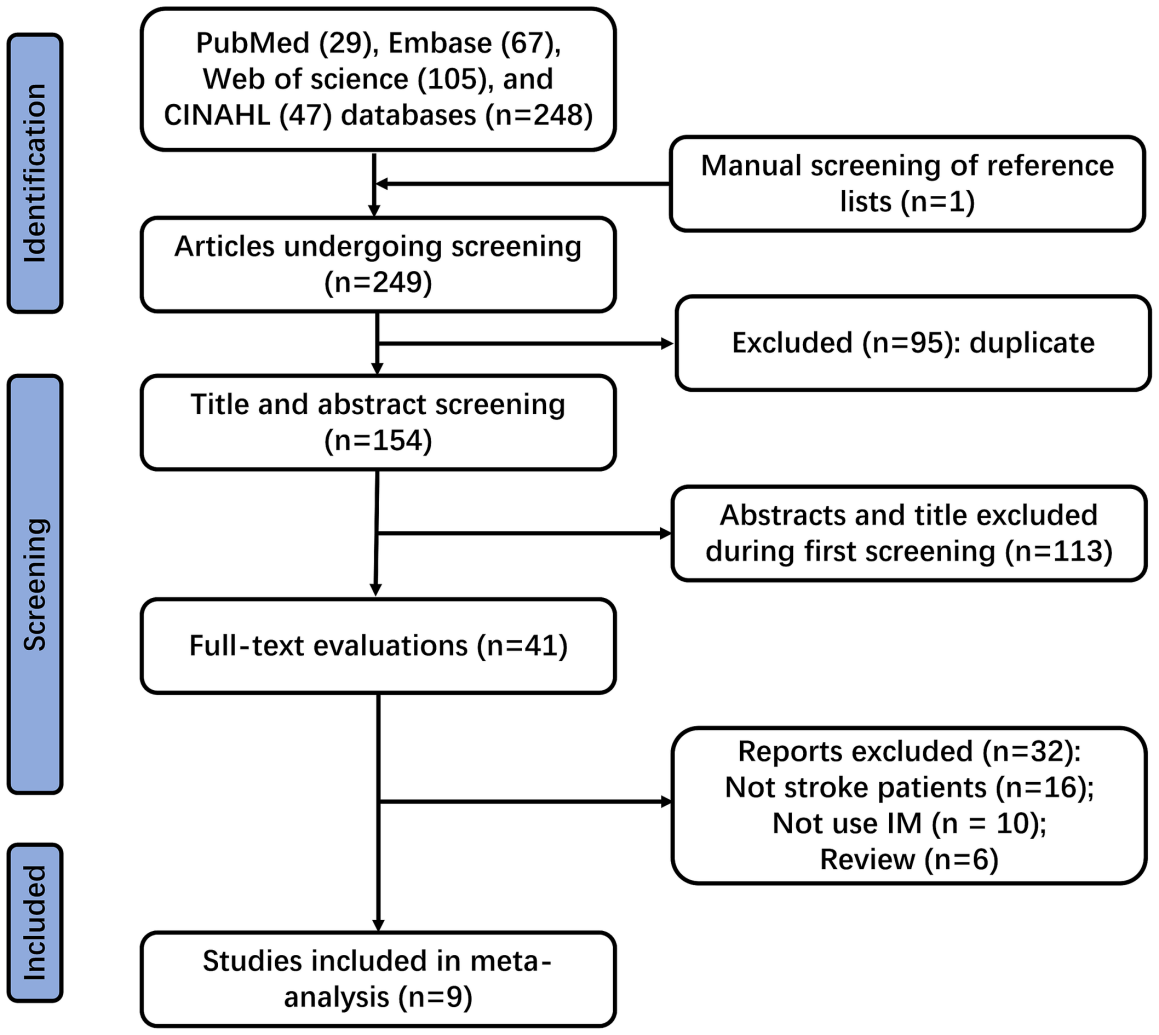
The PRISMA flowchart for literature search and study selection.

A preliminary search yielded 248 relevant records. After removing duplicates, screening titles and abstracts, and conducting full-text reviews, a total of nine studies were ultimately included (12–20). These studies explicitly employed IM to guide the development of behavioral interventions targeting stroke survivors and/or their caregivers. The study selection process is illustrated in the PRISMA flowchart (Figure 1). The key characteristics of the included studies are summarized in Table 1. The majority of the research was conducted in Western countries, predominantly the United States, the United Kingdom, and Canada. We present the characteristics of the nine included studies organized by study type (development-only, development + feasibility, protocol). We then describe IM application patterns, followed by intervention characteristics, feasibility outcomes, and finally clinical outcomes (where available). This organization separates what the evidence shows about development processes from what it shows about effectiveness.

**Table 1:** The characteristics of identified studies.

Study	Country	Study design	Study purpose	Population
Schmid 2010 (12)	USA	An intervention development and implementation study guided by the IM framework. The study describes the systematic process of planning, developing, and preparing for the evaluation of a complex intervention.	To apply the IM framework to guide the systematic development and implementation of an evidence-based, locally tailored secondary stroke prevention program at two specific medical centers	Target population comprised two groups: Intervention providers: Frontline, multidisciplinary clinical providers involved in stroke care across two VAMCs. The needs assessment sample consisted of 44 providers. Intervention recipients: Veteran stroke or TIA survivors and their caregivers. This phase focused on intervention development; therefore, patient outcome data were part of the planned future evaluation.
Sakakibara 2017 (13)	USA	Intervention development study. This paper details the systematic development process of the “Stroke Coach” intervention using the IM protocol.	To describe the application of Intervention Mapping in the systematic development of a theory- and evidence-based intervention—the Stroke Coach—aimed at improving control of lifestyle behavior risk factors in stroke patients.	Target population: Community-dwelling stroke survivors. Planning group: A multidisciplinary planning group of 8 members, including healthcare professionals and researchers with expertise in neurosciences, stroke and cardiac prevention, human nutrition, behavior change theories, self-management, and research methodologies.
Hall 2019 (14)	UK	Intervention development study, applying the first four stages of the IM protocol.	To develop a theory- and evidence-based programme plan for an intervention aimed at reducing burden in carers of stroke survivors.	Intervention target: Carers (caregivers) of stroke survivors. Development Participants: A multi-stakeholder group comprising carers (*n* = 6), researchers (*n* = 3), and health/community service professionals (*n* = 5).
Ezeugwu 2020 (15)	Canada	Intervention development and feasibility study. The paper describes the development of the STUFFS intervention using IM and reports on its initial feasibility, acceptability, and reception.	To describe the systematic development and implementation of a theory- and home-based sedentary behavior change intervention (STUFFS program) for people with stroke.	Intervention target: People with stroke, within 2 weeks of discharge to home from inpatient rehabilitation. Development Participants: A qualitative inquiry with people with stroke (*n* = 13) informed the initial development. The subsequent feasibility evaluation involved 34 participants recruited for the program
Moore 2022 (16)	UK	Systematic intervention development study using the IM framework.	To systematically develop a theory- and evidence- informed multifaceted behavioral intervention targeting physical activity and sedentary behavior in stroke survivors for integration into the stroke rehabilitation care pathway.	Intervention target: Community- dwelling adult stroke survivors. Development Participants: Stroke survivors (*n* = 18 in focus groups; *n* = 21 in consultation workshops). Healthcare Professionals working in stroke services (*n* = 24 in focus groups; *n* = 11 provided feedback via questionnaire)
Auger 2022 (17)	Canada	Protocol for a qualitative, co-design intervention development study, guided by the IM framework.	To co-design, with multiple stakeholders, a theory-driven multifactorial program to improve post-stroke sexual rehabilitation services.	Stakeholders for Co-Design: Persons with stroke (chronic phase), their partners, clinicians, managers, and researchers from five stroke rehabilitation centers in Quebec, Canada.
Denny 2023 (18)	USA	Intervention development and pilot study (formative research). The paper details the use of IM for development and reports preliminary pilot results on usability and short-term outcomes from a previously published trial.	To develop a theory-driven, video-based educational intervention to improve stroke literacy among hospitalized patients with acute stroke.	Target: Acute stroke survivors during hospitalization. Development input: ~50 stroke survivors/family members and a multidisciplinary stroke care team (nurses, physicians, therapists, social workers) for needs assessment. Pilot test: 93 stroke survivors who completed the video intervention (from a larger screened sample of 250).
Wong 2023 (19)	USA	Development study. The paper describes the systematic process of adapting and developing a digital intervention using the IM framework. A separate clinical trial for evaluation is mentioned as future work.	To describe the application of the IM framework and Behavior Change Techniques to adapt an existing face-to-face psychoeducation program into a digital intervention for self-management in people after stroke	Target population: People after stroke. Development Participants: A planning group (*n* = 6) of stakeholders (clinicians, researchers, technology developer, one person with stroke). A separate telephone survey was conducted with people after stroke (*n* = 125) for needs assessment.
Craven 2025 (20)	UK	Development study (Intervention development paper using the Intervention Mapping framework)	To develop a digital, self-guided return-to-work (RTW) toolkit (TTEAM) for stroke survivors and employers in the United Kingdom using the IM approach.	Target population: Working-age stroke survivors and their employers, particularly those in small- or medium-sized organizations with limited access to human resources (HR) or occupational health support. Development Participants: An expert advisory group (*n* = 20) including stroke survivors, healthcare professionals, researchers, HR experts, and charity representatives. Separate online workshops were conducted with employer stakeholders (*n* = 12 total across three workshops).

### Methodological quality

The methodological quality of the included studies was assessed using the EPHPP tool (Table 2). Overall, the global quality ratings of the included studies ranged from “moderate” to “strong.” All nine studies (100%) received “strong” ratings for appropriateness of study design and data collection methods. Regarding selection bias, five studies (56%) were rated “moderate” due to convenience sampling or single-site recruitment; four studies (44%) were rated “strong.” Intervention integrity was rated “strong” in eight applicable studies (100% of those delivering an intervention); Auger, 2022 (17) (protocol paper) was rated “not applicable.” Data analysis clarity was rated “strong” in eight studies (89%) and “moderate” in one study [Denny, 2023 (18), due to use of a novel non-validated questionnaire]. The domains of confounders and blinding were rated “not applicable” for all studies given their developmental aims.

**Table 2:** The methodological quality of included quantitative studies was assessed using the Effective Public Health Practice Project (EPHPP) Quality Assessment Tool.

S/No	Appraisal item	Schmid, 2010 (12)	Sakakibara, 2017 (13)	Hall, 2019 (14)	Ezeugwu, 2020 (15)	Moore, 2022 (16)	Auger, 2022 (17)	Denny, 2023 (18)	Wong, 2023 (19)	Craven, 2025 (20)
1.	Was there any possibility of selection bias?	Moderate	Moderate	Moderate	Strong	Strong	Moderate	Strong	Moderate	Strong
2.	Was the research design appropriate to meet the research objectives?	Strong	Strong	Strong	Strong	Strong	Strong	Strong	Strong	Strong
3.	Have confounders been identified and controlled?	Not applicable	Not applicable	Not applicable	Not applicable	Not applicable	Not applicable	Not applicable	Not applicable	Not applicable
4.	Were participants and/or researchers blinded?	Not applicable	Not applicable	Not applicable	Not applicable	Not applicable	Not applicable	Not applicable	Not applicable	Not applicable
5.	Was the data collection method appropriate for the research?	Strong	Strong	Strong	Strong	Strong	Strong	Strong	Strong	Strong
6.	Were withdrawal/ dropout rates acceptable?	Not applicable	Not applicable	Moderate	Strong	Not applicable	Not applicable	Strong	Not applicable	Not applicable
7.	Was the intervention of acceptable integrity?	Strong	Strong	Strong	Strong	Strong	Not applicable	Strong	Strong	Strong
8.	Was data analysis clear and robust?	Strong	Strong	Strong	Strong	Strong	Strong	Moderate	Strong	Strong

### Application patterns and depth of intervention mapping

All included studies systematically applied the IM framework (Table 3). The application patterns can be categorized into three types: (1) Full application (all six steps): Four studies comprehensively implemented all six steps of IM, extending from initial needs assessment (Step 1) through to the development of implementation and evaluation plans (Steps 5–6) (13, 15, 16, 18); (2) Partial application (focus on development, Steps 1–4): While partial application may reflect an appropriate alignment between research developmental stage and methodological depth, alternative explanations cannot be excluded. Partial application may also result from: (a) journal space constraints limiting reporting of later IM steps; (b) researchers prioritizing development over implementation planning; or (c) methodological drift, where the systematic rigor of early IM steps diminishes in later steps. The current evidence base does not permit differentiation among these explanations. Nevertheless, the observation that only 4/9 studies progressed to formal implementation or evaluation planning (Steps 5–6) identifies a gap in the translational pipeline from intervention development to effectiveness testing (14, 17, 19, 20); (3) Integration with co-design: Two studies deeply integrated the IM process with co-design methodologies, emphasizing the collaborative participation and shared decision-making of multiple stakeholders (e.g., patients, caregivers, clinicians) throughout each phase (17, 20). These categories overlap: Auger, 2022 (17) is counted in both ‘partial application’ (as a protocol applying Steps 1–4) and ‘integration with co-design’; Craven, 2025 (20) similarly applies Steps 1–4 within a co-design framework. Thus, the sum exceeds nine studies because one study (17) satisfies two categorization criteria, and the remaining eight studies are uniquely assigned. No study employed co-design within a full six-step application. One study (12) originally labeled its preliminary needs assessment as ‘Step 0’; for consistency with the classic six-step framework, we have reclassified this needs assessment activity under Step 1 throughout our analysis.

**Table 3:** The summary results for identified studies.

Study	Application of the IM framework	Methods of data collection and analysis	Intervention strategies	Outcome and effect measure
Schmid, 2010 (12)	Fully adhered to the six steps of IM (needs assessment as Step 1, not ‘Step 0’): Step 1 (Needs assessment): Identified provider practices, barriers, needs, and suggestions for delivering secondary stroke prevention via semi-structured interviews (*n* = 44). Step 2 (Matrix of proximal program objectives): Defined behavioral objectives based on guidelines and crossed them with determinants from the Chronic Care Model to create matrices of change objectives. Step 3 (Selection of theory-based methods): Explicitly integrated the Chronic Care Model to structure intervention components and the Theory of Planned Behavior to guide implementation strategies, combined with practical suggestions from the needs assessment. Step 4 (Program design): Designed specific, locally tailored intervention materials and activities based on previous steps. Step 5 (Adoption and implementation plan): Developed an implementation plan involving feedback from local leadership, identification of clinical champions, and creation of program tracking systems. Step 6 (Evaluation plan): Formulated a detailed mixed-methods evaluation plan to assess both process and effect.	Primary data and methods for this development phase: Data collection: Main method: Semi-structured interviews with 44 healthcare providers. Basis: Interview guides were developed based on the Chronic Care Model and clinical guidelines, and were pilot-tested. Data analysis: Thematic analysis of interview transcripts to identify core themes regarding current practices, barriers, needs, and suggestions. Findings provided direct, evidence-based input for subsequent IM steps (particularly Steps 1–3).	The resulting TOOLS program is a multi-level, multi-component intervention strategy, including: Provider/System Level: Education/Training: Training on stroke prevention guidelines, motivational interviewing, and goal-setting techniques. Decision support tools: Development and use of a stroke risk factor “prescription pad,” checklist posters, and standardized patient education materials. Workflow integration: Incorporating stroke prevention goals into rehabilitation notes, standardizing discharge processes. Patient level: Self-management support: Teaching goal-setting techniques for behavior change via trained providers. Resource linking: Systematic referral to existing VA services via the prescription pad. Social support: Establishing stroke support groups and introducing a “peer-to-peer” support program. Core feature: Emphasis on local tailoring, with intervention components adapted based on the distinct needs and resources of the two sites.	Planned Primary Evaluation Dimensions: Implementation Process outcomes: Assessed via medical record review for provider behavior change. Patient outcomes: Assessed via surveys and medical record review, including: Health-related quality of life: Stroke-Specific Quality of Life scale. Clinical and Behavioral measures: Physical function, depressive symptoms, self-efficacy, stroke-related knowledge and beliefs. Risk Factor Management. Assessment Time points: Planned at baseline, 3 months, and 6 months post-intervention.
Sakakibara, 2017 (13)	Fully adhered to the six steps of IM: Step 1 (Needs Assessment): Established and worked with a multidisciplinary planning group; Conducted a detailed literature review to understand issues in secondary stroke prevention and conceptualize the intervention framework. Step 2 (Proximal Intervention Objectives): Identified lifestyle behaviors to change; Identified theoretical determinants of those behaviors as the proximal objectives. Step 3 (Theory-Based Methods and Practical Strategies): Identified evidence-based intervention methods targeting proximal objectives; Translated methods into three core practical delivery strategies: Lifestyle Coaching, Self-management Manual, Self-monitoring Kit. Step 4 (Program design): Conceptualized and integrated the: Dose; Delivery Mode; Organization; Stakeholder Feedback and Revision. Step 5 (Implementation plan): Developed initial implementation materials including training presentations and a coaching manual, and planned methods to assess and ensure intervention fidelity. Step 6 (Evaluation plan): Developed a research protocol for a multi-site, single-blind randomized controlled trial to evaluate the Stroke Coach on primary and multiple secondary outcomes.	Primary data and methods for this development phase: Data collection: Main Methods: Literature review and input/ feedback from the planning group and stakeholders. Specific Activities: Ongoing discussions and decision-making within the planning group; presentation of preliminary program concepts to stakeholders for feedback and revision. Data analysis: This phase involved qualitative synthesis and decision-making, iteratively revising the intervention based on planning group expertise and stakeholder feedback, without formal data analysis.	The resulting “Stroke Coach” is a patient-centered, community-based, telehealth-delivered chronic disease management intervention. Core strategies include: Lifestyle coaching: One-on-one, patient-centered telephone coaching by trained health workers using motivational interviewing and the “5A” model to facilitate behavior change and self-management. Self-management manual: Provides detailed information on self-managing lifestyle behaviors for stroke risk control, serving as a discussion document and resource during coaching. Self-monitoring kit: Includes a Health Report Card, pedometer, blood pressure monitor, tape measure, food/physical activity diaries, etc., to help participants monitor behaviors/ physiological indicators and track progress. The Health Report Card grades participants’ risk factors from A to F based on clinical guidelines and is central to coaching discussions. Theoretical foundation: Social Cognitive Theory as the core premise for behavior change, with Control Theory methods directed toward sustaining long-term change. Intervention methods were explicitly linked to theoretical determinants and practical strategies via a matrix.	Planned primary hypothesis and outcome: Improvement in a global measure of lifestyle behavior assessed by the Health-Promoting Lifestyle Profile II. Planned Secondary Outcomes: Specific lifestyle behaviors: Physical activity, diet, etc. Psychological and health status: Depressive symptoms, health-related quality of life, cognitive function. Cardiovascular risk: Cardiovascular risk indicators. Process and Experience evaluation: Post-intervention exit surveys and a qualitative study to assess participant experiences, satisfaction, and coaches’ perceptions and fidelity of intervention delivery.
Hall, 2019 (14)	Applied Stages 1 to 4 of IM: Stage 1 (Needs assessment): Integrated evidence from three components: a review of reviews, a systematic review of qualitative studies, and 33 semi-structured interviews to build a “logic model of burden.” Stakeholders prioritized the key need: “Carers need to feel prepared before, and during, the transition from hospital to home.” Stage 2 (Objectives): Defined behavioral/environmental outcomes and performance objectives. The Theoretical Domains Framework was used to identify theoretical determinants for creating matrices of change objectives. Stage 3 (Methods and strategies): Collaborated with stakeholders to generate intervention ideas and selected theory-based methods and practical applications for the change objectives. Stage 4 (Programme plan): Produced the organized plan for the “Preparing is Caring” intervention, consisting of training packages for support providers and multiple components for carers.	Data for development: Synthesized evidence from two systematic reviews and collected primary qualitative data via 33 interviews with carers. Systematically captured stakeholder input through 5 facilitated meetings. Analysis: Conducted thematic synthesis of review evidence and thematic analysis of interview data. Stakeholder meeting outputs were recorded and integrated.	“Preparing is Caring” is a multi-component, multi-level intervention: For professionals: A training package (5 core modules plus ongoing supervision) to change how they support carers. For carers: Elements delivered by a “single point of contact” before, during, and after the hospital-to-home transition, including face-to-face discussions, an “in case of” plan, key contact cards, and helpline access.	Planned future work: The paper explicitly states that future research is required to implement and evaluate the intervention (which corresponds to IM Stages 5 and 6). The development process yielded a detailed programme plan, logic models, and a theoretical foundation for future evaluation.
Ezeugwu, 2020 (15)	Applied all six steps of IM: Steps 1–4 (Development): Needs assessment via literature review and qualitative study (*n* = 13); definition of program objectives and matrices of change (Table 1); selection of theory-based methods (Social Cognitive Theory) and practical applications (Table 2); program production including a refined program manual. Steps 5–6 (Implementation and evaluation): Adoption plan via clinician collaboration; process and outcome evaluation in a feasibility study (*n* = 34) assessing satisfaction (89%) and thematic analysis of exit interviews (*n* = 25)	Development phase: Qualitative interviews (*n* = 13) to explore perceptions, awareness, and barriers/facilitators. Feasibility/Evaluation phase: Mixed methods: Quantitative satisfaction scores; Qualitative exit interviews (*n* = 25) analyzed using thematic content analysis. Objective activity monitoring was used but results are not the focus of this development paper	STUFFS program: An 8-week, home-based, therapist-delivered program. Core components: Education: On health risks of prolonged sedentary behavior. Behavior change strategies: Action planning, goal setting, breaking up sitting. Self-monitoring and motivation: Use of a consumer wearable activity monitor (Misfit) for real-time feedback and as a motivational tool.	Primary focus of this paper: Feasibility, acceptability, and program reception. Quantitative: High participant satisfaction (average 89%). Qualitative (Themes): Facilitators (self-monitoring, follow-up support, health benefits, dyad-focused approach, habit formation) and Barriers(fatigue, memory issues, fear of falls)
Moore, 2022 (16)	Applied all six steps of IM comprehensively: Step 1 (Needs assessment): Combined a systematic review, qualitative focus groups with stroke survivors/HCPs, and a review of local care pathways. Step 2 (Objectives): Defined behavioral outcomes, performance objectives, and change objectives for both stroke survivors and HCPs, mapped to the Theoretical Domains Framework. Step 3 (Theory-based methods): Selected behavioral change theories and linked TDF domains with specific Behavior Change Techniques. Step 4 (Program development): Developed prototype intervention (PARAS) and refined it through iterative consultation workshops with stroke survivors and feedback from HCPs. Step 5 (Implementation plan): Formulated a detailed plan using the TIDieR checklist and APEASE criteria to ensure real-world applicability. Step 6 (Evaluation plan): Designed a feasibility study protocol.	Mixed-Methods Approach: Systematic Review of existing RCTs. Qualitative Focus Groups and Workshops: Audio-recorded, transcribed, and analyzed using the Theoretical Domains Framework for thematic analysis. Questionnaires for HCP feedback. Pathway Review via questionnaires to community stroke teams.	PARAS (Physical Activity Routines After Stroke): A multi-faceted, supported self-management intervention. For stroke survivors: Toolkit including a workbook, local activity repository, self-monitoring tools (diary, pedometer), goal-setting aids. Delivered via at least two sessions (initial face-to-face, follow-up flexible) by a trained HCP. For healthcare professionals: A training programme to develop behavior change counseling skills, including a manual and face-to-face training.	This is a development paper; no effectiveness outcomes are reported. Primary output: A fully developed intervention plan (PARAS) and a protocol for a feasibility study. Process outcomes: High acceptability of prototype materials reported by stroke survivors (>75% found workbook easy to use) and HCPs in feedback questionnaires.
Auger, 2022 (17)	The protocol plans to apply the first four steps of IM, integrated within a four-phase co-design methodology: Step 1. Needs Assessment (Co-design Exploration): Using an adapted online LEGO® Serious Play® method with clinicians and work groups to understand barriers and needs. Step 2. Preparing Matrices of Change (Co-design): Involving an advisory committee to define change objectives based on needs assessment data. Step 3. Selecting Methods and Strategies (Co-design/Validation): Using the Behavior Change Wheel and advisory/work groups to select and validate behavior change strategies. Step 4. Producing the program (Development): Developing the final program components described using the TIDieR checklist	Planned Methods: Data collection: Facilitated online group activities. Sessions will be audio-recorded. Data analysis: Semi-deductive thematic analysis of transcripts by two independent reviewers, using the Theoretical Domains Framework and the ICF Core Set for Stroke as guiding frameworks. Analysis will be validated iteratively with stakeholder groups	Not Yet Developed. The outcome of this protocol will be a multifactorial program plan. The content will be determined through the co-design process. It is intended to target multiple stakeholders (patients, partners, clinicians, managers) and levels of the environment to change practice.	Not Applicable (Protocol). The primary output of the planned study is a co-designed program plan, not an evaluated intervention. The protocol mentions that future steps (evaluation) will be needed to assess the program’s feasibility and effectiveness.
Denny, 2023 (18)	Applied all six steps of IM: Step 1. Needs assessment: Literature review + interviews with survivors, families, and care team. Step 2. Objectives: Defined short, medium, long-term outcomes and change objectives using a logic model. Step 3. Theory and methods: Selected the McGuire Communication Persuasion Matrix to guide video design. Step 4. Program development: Created a 5-min educational video with animations, diverse cast, and a 10-item evaluation questionnaire. Step 5. Implementation plan: Developed procedures for video delivery at bedside, staff training, and screening. Step 6. Evaluation plan: Used the developed questionnaire to assess knowledge, self-efficacy, and satisfaction pre-, post-, and 30-days post-video.	Development phase: Qualitative needs assessment via stakeholder interviews. Pilot evaluation phase: Quantitative assessment using a novel 10-item questionnaire measuring stroke knowledge (8 items), self-efficacy (1 item), and satisfaction (1 item). Administered pre-, post-, and 30-days post-intervention. Statistical comparison of scores (Wilcoxon signed-rank test).	“Stroke Literacy Video”: A 5-min, video-based, standalone educational intervention. Content: Defines stroke, teaches FAST acronym, explains risk factors and prevention, emphasizes calling 911 and follow-up care. Design Principles: Theory-driven (CPM), uses visuals/animations, diverse cast, 6th-grade reading level, later added Spanish subtitles. Delivery: Shown once at bedside on a laptop prior to hospital discharge.	Pilot results: Stroke knowledge: Median score increased from 6/8 pre-video to 7/8 post-video (*p* < 0.001), maintained at 30 days (*p* = 0.04). Self-efficacy: Proportion “very certain” in recognizing stroke symptoms increased from 35.5 to 53.5% post-video, maintained at 30 days. Satisfaction: Proportion “very satisfied” with education increased from 49.5 to 74.2% post-video
Wong, 2023 (19)	The study explicitly used the first four steps of the IM process: Step 1 (Needs assessment): Formed a planning group, conducted a telephone survey (*n* = 125), and performed a systematic literature review. Step 2 (Matrix of change objectives): Identified behavioral determinants and performance objectives using the COM-B model. Step 3 (Theory-based methods and practical strategies): Selected Mechanisms of Action and linked them to specific Behavior Change Techniques to design intervention components. Step 4 (Program development): Developed the iSMART intervention materials with iterative feedback from the planning group, who assessed acceptability, appropriateness, and feasibility.	Data collection: Mixed methods during development: telephone survey, systematic review, planning group feedback (qualitative), and completion of validated implementation measures. Analysis: Descriptive statistics for survey and feasibility measures. Meta-analytic techniques for the systematic review. Thematic synthesis/consensus for planning group feedback	A 12-week digital intervention with three core components: Psychoeducation Group Sessions (via videoconference): Adapted from IPASS, teaching self-management skills. Individual Behavioral Coaching (via videoconference): Based on Behavioral Activation principles, focusing on collaborative goal setting and barrier management. SMS text messaging: Automated and tailored messages for goal reminders, tips, motivation, and mood check-ins, managed via a clinician dashboard.	Primary utcomes: Acceptability, Appropriateness, and Feasibility of the developed iSMART intervention. Measures: Validated 4-item scales (AIM, IAM, FIM) rated on a 5-point Likert scale, completed by the planning group. Results: High scores were reported: Acceptability (mean 4.63, SD 0.38), Appropriateness (mean 4.63, SD 0.38), Feasibility (mean 4.58, SD 0.34).
Craven, 2025 (20)	The study explicitly followed Steps 1–4 of the six-step IM process: Step 1 (Logic model of the problem): Conducted a mixed-methods needs assessment and held Workshop 1 with stakeholders to refine the problem model and set the intervention goal. Step 2 (Logic model of change): Defined behavioral outcomes, performance objectives, and determinants. Created matrices of change and a logic model of change, informed by advisory group feedback. Step 3 (Intervention design): Selected theory- and evidence-based behavior change methods and practical applications. Workshop 2 informed the scope, sequence, and components of the TTEAM toolkit. Step 4 (Intervention production): Developed the TTEAM prototypes. Conducted pretesting via task-based review and discussions with the advisory group (*n* = 15: 7 stroke survivors, 4 employers, 4 professionals) and held Workshop 3 for further feedback.	Data collection: Mixed methods: Needs assessment, stakeholder workshops, and pretesting. Analysis: For pretesting feedback, framework analysis was used to structure feedback based on constructs from the Technology Acceptance Model, System Usability Scale, and ICF.	Format: Two separate, self-guided, interactive eLearning packages (Xerte) for stroke survivors and employers. Content: Five-step process covering: (1) Readiness/Stroke and Communication, (2) Appraisal of needs/Employer Roles, (3) Disclosure of needs/Understanding employee needs, (4) Planning adjustments, (5) Ongoing review. Components: Theory-based educational content, interactive activities, and downloadable PDF tools. Theory: Incorporates multiple behavior change methods linked to specific performance objectives.	Primary outcome: The developed TTEAM toolkit prototype and its perceived acceptability, usability, and usefulness. Measures: Qualitative feedback from the advisory group and workshop participants, structured using framework analysis. No quantitative scores from validated scales are reported in the results. Results: Pretesting indicated the toolkit was perceived as comprehensive, empowering, useful, and fostering open communication. Minor refinements and technical improvements were suggested.

### Developed intervention strategies and core characteristics

The interventions developed through IM were complex and multifaceted, exhibiting the following core characteristics: (1) Theory-driven: All interventions explicitly integrated at least one behavior change theory or framework as the foundation for selecting change methods and practical applications; (2) Multi-level and multi-component: Interventions commonly targeted multiple socio-ecological levels, including individual, interpersonal, and organizational factors. For instance, the “TOOLS” project by Schmid et al. concurrently incorporated clinician training, clinical decision support tools, and patient self-management support (12); (3) Diverse modalities: Intervention delivery formats included face-to-face individual or group counseling (13), home-based telehealth coaching (15), digital health toolkits (19, 20), and brief video-based education (18); (4) Contextualization and user engagement: The majority of interventions emphasized tailoring to specific settings (12) or user groups (20), incorporating iterative refinements based on continuous stakeholder feedback.

### Preliminary efficacy and feasibility

Among the six studies that included feasibility or pilot evaluations, the interventions demonstrated strong acceptability, feasibility, and preliminary positive signals. (1) Acceptability and feasibility: Quantitative ratings were generally high. For example, Wong et al. reported mean scores for acceptability, appropriateness, and feasibility all exceeding 4.5 out of 5 (19). Qualitative feedback corroborated the interventions’ relevance, practicality, and ease of use; (2) Knowledge, beliefs, and self-efficacy: Denny et al.’s video-based intervention significantly enhanced stroke survivors’ disease-related knowledge and strengthened their self-efficacy in recognizing stroke symptoms (18); (3) Behavioral intentions and process indicators: Several studies, utilizing qualitative interviews or process evaluations, reported participants’ positive behavioral intentions and initial attempts regarding self-management, physical activity, or the interruption of sedentary behavior; (4) Limitations: Currently, no studies have reported long-term, hard clinical outcomes (e.g., recurrent stroke rates, mortality) or large-scale effectiveness data. Existing evidence of effectiveness is primarily derived from self-reported measures and short-term follow-ups.

To enhance analytical clarity, we stratified findings across three distinct domains. First, development outputs (*n* = 9): all included studies produced logic models and intervention manuals, though reporting transparency varied. Second, feasibility outcomes (*n* = 6): among studies that conducted feasibility or pilot evaluations, interventions demonstrated high acceptability (mean ratings >4.5/5 where reported), appropriateness, and feasibility. Third, clinical outcomes (*n* = 0): no study reported long-term hard clinical outcomes. Two studies reported short-term, self-reported behavioral outcomes at follow-up periods of 30 days or less. This stratification clarifies that the current evidence base supports claims about development processes and feasibility only, not clinical effectiveness.

### Key outputs and challenges in the development process

All included studies produced structured logic models and detailed intervention manuals, specifying the intervention’s goals, components, implementation procedures, and theoretical rationale. These outputs lay a crucial foundation for subsequent rigorous evaluation and dissemination. However, the transparency of reporting regarding the application of specific IM steps—particularly Step 2 (Program Objectives and Matrices) and Step 3 (Theory-Based Methods and Practical Strategies)—was suboptimal in some studies. This lack of detailed description somewhat compromises the reproducibility of the methodological processes employed. To provide explicit comparative synthesis of how studies operationalized these critical steps, Supplementary File 2 details the determinants, theoretical frameworks, matrix reporting practices, and implementation strategies across all nine included studies.

To systematically characterize reporting transparency, we quantified the adequacy of reporting for each IM step across all nine included studies (Table 4). Reporting was considered “adequate” if the study provided sufficient detail to allow replication of that specific step (e.g., explicit matrix table for Step 2; named theory-based methods linked to practical strategies for Step 3). Step 1 (needs assessment) was adequately reported in all nine studies (100%). Step 4 (intervention production) was adequately reported in eight studies (89%). However, Step 2 (matrices of change objectives) was adequately reported in only two studies [22%; Ezeugwu, 2020 (15) and Moore, 2022 (16), both of whom provided explicit matrix tables or Supplementary materials]. Step 3 (theory-based methods and practical strategies) was adequately reported in five studies (56%); the remaining four studies named theoretical frameworks but did not clearly specify the logical chain linking theoretical methods to practical applications. This reporting gap substantially constrains methodological replicability and theoretical accumulation in the field.

**Table 4:** Reporting adequacy of intervention mapping steps across included studies (*n* = 9).

IM Step	Description	Adequately reported (*n*, %)	Studies with adequate reporting
Step 1	Needs assessment	9 (100%)	All studies
Step 2	Matrices of change objectives	2 (22%)	Ezeugwu, 2020 (15); Moore, 2022 (16)
Step 3	Theory-based methods and practical strategies	5 (56%)	Schmid, 2010 (12); Sakakibara, 2017 (13); Ezeugwu, 2020 (15); Moore, 2022 (16); Wong, 2023 (19)
Step 4	Intervention production	8 (89%)	All except Auger, 2022 (17) (protocol)
Step 5	Implementation plan	4 (44%)	Schmid, 2010 (12); Sakakibara, 2017 (13); Ezeugwu, 2020 (15); Moore, 2022 (16)
Step 6	Evaluation plan	5 (56%)	Schmid, 2010 (12); Sakakibara, 2017 (13); Ezeugwu, 2020 (15); Moore, 2022 (16); Denny, 2023 (18)

**Figure 2:**
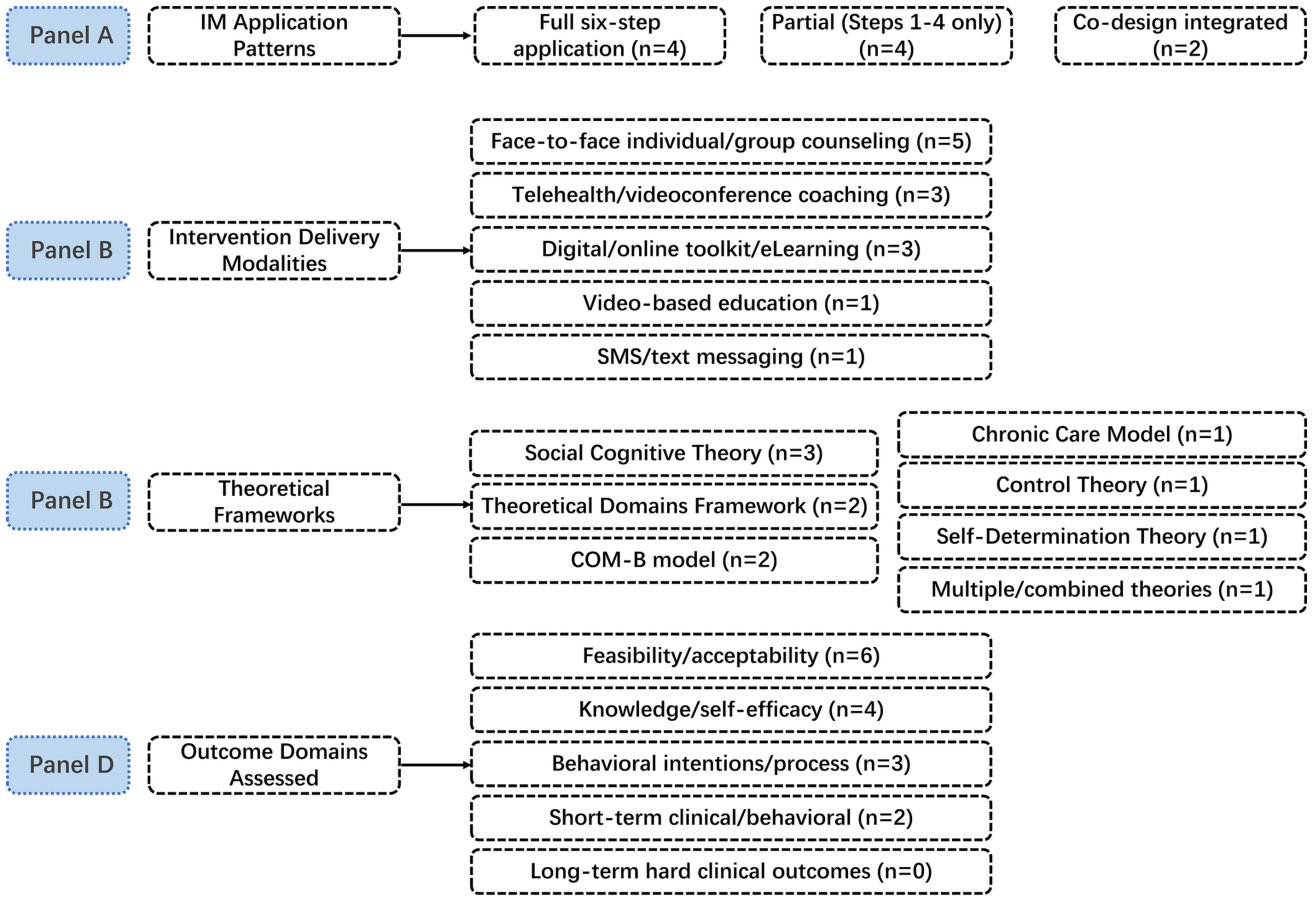
Visual synthesis of intervention mapping application patterns, delivery modalities, theoretical frameworks, and outcome domains across nine included studies. **(A)** Distribution of full six-step IM application (*n* = 4), partial application (Steps 1–4 only, *n* = 4), and co-design integration (*n* = 2). **(B)** Intervention delivery modalities: face-to-face (*n* = 5), telehealth (*n* = 3), digital/online (*n* = 3), video-based (*n* = 1), SMS (*n* = 1). **(C)** Theoretical frameworks employed: Social Cognitive Theory (*n* = 3), Theoretical Domains Framework (*n* = 2), COM-B (*n* = 2), Chronic Care Model (*n* = 1), Control Theory (*n* = 1), Self-Determination Theory (*n* = 1), multiple theories (*n* = 3). **(D)** Outcome domains assessed: feasibility/acceptability (*n* = 6), knowledge/self-efficacy (*n* = 4), behavioral intentions (*n* = 3), short-term clinical/behavioral (*n* = 2), long-term hard clinical outcomes (*n* = 0). See main text for study citations and full descriptions.

Figure 2 provides a visual synthesis of the key characteristics across all nine included studies, organized into four domains: (A) IM application patterns (full six-step vs. partial vs. co-design-integrated); (B) intervention delivery modalities (face-to-face, telehealth, digital, video); (C) theoretical frameworks employed (Social Cognitive Theory, TDF, COM-B, Chronic Care Model, etc.); and (D) outcome domains assessed (feasibility/acceptability, knowledge/self-efficacy, behavioral intentions, clinical outcomes). This figure illustrates the predominance of partial IM applications, the diversity of delivery modalities, the concentration on individual-level behavioral theories, and the near-absence of long-term clinical outcome assessment across the current evidence base.

## Discussion

This study systematically reviewed the current state of research employing the IM framework to guide the development of behavioral interventions for stroke survivors. To the best of our knowledge, based on systematically searched English-language peer-reviewed literature, this is the first integrated analysis of methodological application patterns, intervention characteristics, and preliminary effects in this field. Through in-depth analysis of nine included studies, this review elucidates the diversity, core advantages, and existing challenges of IM applications in stroke rehabilitation, providing an empirical foundation for the optimized utilization and reporting standardization of this methodology.

Our primary finding is that IM application in stroke rehabilitation follows three patterns: full six-step application (4/9, 44%), partial application focused on Steps 1–4 only (4/9, 44%), and integration with co-design methodologies (2/9, 22%). Fewer than half of the included studies progressed to formal implementation or evaluation planning (Steps 5–6). This pattern should not be interpreted as methodological deficiency. Most behavioral intervention studies in stroke are situated in early-to-middle steps of intervention development, where the central question is “how can we design a scientifically sound, acceptable, and contextually appropriate intervention protocol?” rather than “does this intervention work in a large-scale trial?” Consequently, researchers concentrating resources on Steps 1 through 4 reflect a dynamic alignment between research developmental stage and methodological application depth. However, alternative explanations cannot be excluded. Partial application may also result from: (a) journal space constraints limiting reporting of later IM steps; (b) researchers prioritizing development over implementation planning; or (c) methodological drift, where the systematic rigor of early IM steps diminishes. The current evidence does not permit differentiation among these explanations. Regardless of cause, the observation that only 4/9 studies included implementation or evaluation planning identifies a translational gap: rigorously developed interventions (Steps 1–4) may stagnate at the feasibility stage without progressing to effectiveness trials (21). The two studies that integrated IM with co-design methodologies (17, 20) represent a notable methodological evolution. Traditional IM emphasizes stakeholder participation primarily as “consultative”—researchers lead framework construction while stakeholders provide feedback at predetermined junctures (6). Co-design reconstructs this relationship into “co-creative” collaboration, granting patients, caregivers, and frontline clinicians equal methodological voice (22). This integration may help address the implementation challenges that laboratory-developed interventions face in real-world contexts. However, current integration remains exploratory; how to incorporate co-design flexibility without compromising IM structural rigor requires further methodological research.

All nine studies explicitly cited at least one behavior change theory (12–20). Social Cognitive Theory, the Theoretical Domains Framework (TDF), and COM-B appeared most frequently (13, 14, 16, 19). At face value, this finding highlights IM’s advantage over traditional intervention development approaches: IM compels researchers to explicitly link interventions to theoretical foundations (6, 9). However, citation of a theory should not be conflated with theoretical fidelity (23). In four of the nine studies, the logical chain from theoretical constructs to practical strategies was not transparently reported (Table 4). Readers could determine that a theory was named but could not determine whether that theory genuinely guided intervention design or was cited *post hoc*. For example, a study might list TDF as a framework without specifying which domains targeted which intervention components or how domain-specific barriers were addressed through particular behavior change techniques (14, 16). Thus, while IM facilitates theory integration, the current evidence base does not permit conclusions about the quality or depth of theoretical application in stroke behavioral interventions. Future studies should explicitly map each practical strategy to a specific theoretical method and, in turn, to a targeted determinant, using the matrix structure that IM provides (6). This is not an optional reporting enhancement but a core requirement for theoretical accumulation in the field.

IM-guided stroke interventions were consistently multi-level (targeting patients, caregivers, and healthcare providers), multi-component (typically 3–5 active ingredients), and contextually tailored. This complexity aligns with the multidimensional nature of post-stroke behavioral determinants, which include individual knowledge and beliefs, caregiver support, healthcare access, and environmental factors (3, 23). Among the six studies that included feasibility or pilot evaluations, interventions demonstrated high acceptability, appropriateness, and feasibility (13, 15, 16, 18–20). Quantitative ratings were generally high (19). Qualitative feedback corroborated that interventions were perceived as relevant, practical, and easy to use (15, 18). However, the review found no evidence regarding clinical effectiveness. No study reported long-term hard clinical outcomes (e.g., recurrent stroke rates, mortality, major adverse cardiovascular events). Existing effectiveness data derive exclusively from self-report measures and short-term follow-ups (30 days or less in the one study reporting pre-post comparisons) (18). Acceptability and feasibility are necessary preconditions for effectiveness but are not substitutes for it (24). This evidentiary gap reflects a shared challenge within stroke behavioral intervention research: the trajectory from intervention development to feasibility validation to efficacy confirmation is protracted and resource-intensive (5, 21). Nevertheless, the gap serves as an important caution: IM’s ultimate value as an intervention development methodology should be demonstrated through improvements in patients’ long-term health outcomes, not termination at favorable process indicators.

A central finding of this review is the systematic deficiency in reporting of two critical IM steps. Step 2 (matrices of change objectives) was adequately reported in only 2 of 9 studies (22%). Step 3 (theory-based methods and practical strategies) was adequately reported in 5 of 9 studies (56%). In contrast, Step 1 (needs assessment) was adequately reported in all nine studies, and Step 4 (intervention production) in eight studies. Matrix reporting opacity has concrete consequences. Without explicit matrices, external researchers cannot trace the logical inference chain from needs assessment to intervention components. A reader cannot determine why a particular strategy was selected, which determinant it targets, or how success would be measured. This undermines replicability, limits theoretical accumulation, and prevents systematic adaptation of interventions to new contexts (6, 25). Multiple factors may explain this gap: journal space constraints may incline researchers toward condensing methodological details; some researchers may lack sufficient understanding of IM technical operations; or matrix construction may not have been systematically executed during the actual development process. Regardless of etiology, we recommend that journals and reviewers require explicit matrix reporting (as tables in text or Supplementary materials) for studies claiming to use IM (26, 27).

Behavioral interventions developed in one cultural context cannot be assumed transferable to another without systematic adaptation. This challenge is particularly salient for stroke rehabilitation, where beliefs about illness, family involvement patterns, healthcare-seeking behaviors, and acceptable therapist-patient communication styles vary substantially across cultures (28). Encouragingly, the IM framework provides structured mechanisms for cultural adaptation through Step 1 (needs assessment with local stakeholders) and Step 2 (matrix construction that can incorporate culturally-specific determinants). Among our included studies, Schmid et al. (12) explicitly emphasized ‘local tailoring’ across two Veterans Health Administration sites, adapting intervention components based on site-specific resources and provider practices. However, no included study addressed cross-national or cross-ethnic cultural adaptation. We propose three practical strategies for future IM-guided cultural adaptation: (a) conduct preliminary qualitative research with local stroke survivors and family caregivers to elicit culturally-specific barriers and facilitators; (b) involve community stakeholders (e.g., religious leaders, traditional healers where relevant) in the planning group for Steps 1–3; and (c) pilot-test adapted interventions with cognitive interviewing to ensure that behavior change methods (e.g., ‘role modeling,’ ‘persuasive communication’) are culturally congruent. Without such adaptations, effective interventions may fail upon translation to new cultural contexts, wasting development investments and leaving patient needs unmet.

Notably, none of the included studies explicitly measured patient satisfaction with reintegration outcomes (e.g., return-to-work satisfaction, family role adjustment, social participation fulfillment) as a primary or secondary endpoint. This gap is consequential: a stroke survivor may achieve excellent blood pressure control and medication adherence (traditional behavioral targets) yet remain profoundly dissatisfied with their quality of life and social participation. The IM framework is well-suited to address this gap because Step 2 (matrices of change objectives) could explicitly incorporate ‘satisfaction with reintegration’ as a change objective, with corresponding performance objectives such as ‘patient identifies personally meaningful social roles’ and ‘patient expresses confidence in resuming family activities.’ Future IM-guided interventions should systematically integrate patient satisfaction and reintegration quality as co-primary outcomes alongside traditional clinical and behavioral measures.

Three directions are priorities. First, future IM-guided studies must adhere to transparent reporting standards for Steps 2 and 3. Explicit matrix tables and theory-to-strategy mappings should be provided as Supplementary materials or in text tables (26, 27). Second, the field requires large-sample, long-term, multi-center trials with hard clinical endpoints (stroke recurrence, cardiovascular events, mortality) to determine whether IM-guided interventions improve patient outcomes, not just feasibility indicators (24). Third, cross-cultural adaptation and validation of IM-guided interventions in non-Western healthcare systems and low-resource settings is needed, as all included studies were conducted in high-income Western countries (USA, UK, Canada) (28).

This review has several limitations. First, only English-language publications were included. Stroke rehabilitation research has expanded substantially in non-English speaking regions (China, Japan, South Korea, Brazil), where IM may have been applied in local-language publications. The extent of this bias is difficult to estimate, but we acknowledge it as a limitation (29). Second, due to substantial heterogeneity among included studies regarding populations, interventions, and outcomes, narrative synthesis rather than meta-analysis was used, limiting effect estimate precision. Third, the EPHPP tool’s “not applicable” ratings for confounders and blinding compressed the presentation of between-study quality variation. Fourth, the review focused on published literature without systematically capturing ongoing studies or negative results, potentially introducing publication bias. Fifth, the small number of included studies (*n* = 9) limits the generalizability of findings. Sixth, the EPHPP quality ratings reflect methodological rigor within developmental aims, not low risk of bias for causal effect estimation.

## Conclusion

This systematic review answered its primary aim by identifying three distinct patterns of IM application in stroke behavioral intervention development (full six-step, partial Steps 1–4, and co-design integration). Regarding secondary aims, the review characterized IM-guided interventions as theory-informed, multi-level, and contextually tailored, but also identified substantial variability in reporting transparency, particularly for Step 2 (matrices of change objectives) and Step 3 (theory-to-strategy translation). The evidence base does not permit conclusions about whether IM-guided interventions improve clinical outcomes, as no study reported such endpoints. Current evidence supports claims about feasibility, acceptability, and development processes only.

For researchers considering IM, this review offers three practical recommendations: (1) prioritize transparent reporting of Step 2 matrices and Step 3 theoretical methods, using tables or Supplementary materials; (2) explicitly plan for the transition from development (Steps 1–4) to implementation and evaluation (Steps 5–6); and (3) when resources are constrained, partial application focused on rigorous needs assessment and matrix construction may be preferable to superficial coverage of all six steps. Future research should advance mature IM-developed interventions into large-sample, long-term, multi-center trials with hard clinical endpoints. Without such evidence, the ultimate value of IM for improving stroke patient outcomes remains undemonstrated.

## Data Availability

The original contributions presented in the study are included in the article/Supplementary material, further inquiries can be directed to the corresponding author.
