# Supplementary material for: A systematic review on using intervention mapping to guide behavioral interventions for improving stroke patient outcomes
Source: Front Med (Lausanne). 2026 Jun 29;13:1850511. doi: 10.3389/fmed.2026.1850511 (PMC13357211; doi:10.3389/fmed.2026.1850511)
Supplement: Supplementary file 1 [file Data_Sheet_1.DOCX]

Table S1. Operationalization of IM Step 2 (Change Objectives Matrices) and Step 3 (Theory-Based Methods & Practical Strategies)

| Study | Step 2: Determinants Targeted | Step 2: Matrix Explicitly Reported? | Step 3: Behavior Change Theory/Framework | Step 3: Key Practical Strategies |
| --- | --- | --- | --- | --- |
| Schmid 2010 [12] | Provider-level: knowledge, skills, self-efficacy, outcome expectations; System-level: resources, workflow | No (described narratively) | Chronic Care Model; Theory of Planned Behavior | Provider training; decision support tools (prescription pad, posters); workflow redesign |
| Sakakibara 2017 [13] | Lifestyle behaviors (physical activity, diet); Self-efficacy; Outcome expectations | No (described narratively) | Social Cognitive Theory; Control Theory | Telephone lifestyle coaching; self-management manual; self-monitoring kit (pedometer, BP monitor, diaries) |
| Hall 2019 [14] | Carer preparedness; Knowledge; Skills; Social support | No (described narratively) | Theoretical Domains Framework (TDF) | Training package for professionals (5 modules); single point of contact for carers; "in case of" plans |
| Ezeugwu 2020 [15] | Sedentary behavior; Self-efficacy; Goal-setting skills; Outcome expectations | Yes (provided as table in original) | Social Cognitive Theory | Education on sedentary risks; action planning; wearable activity monitor (Misfit) for self-monitoring |
| Moore 2022 [16] | Physical activity; Sedentary behavior; Provider skills in behavior change counseling | Yes (provided as supplementary material) | TDF; Self-Determination Theory; Behavior Change Techniques (BCTs) | Stroke survivor toolkit (workbook, pedometer, diary); HCP training program in BCTs |
| Auger 2022 [17] | Sexual rehabilitation barriers (patient, partner, clinician, system levels) | Planned (protocol only) | TDF; ICF Core Set for Stroke; Behavior Change Wheel | Protocol stage – LEGO® Serious Play® workshops planned for co-design |
| Denny 2023 [18] | Stroke knowledge; Symptom recognition self-efficacy | No (simplified logic model provided) | McGuire Communication Persuasion Matrix | 5-minute educational video; bedside delivery; 10-item knowledge questionnaire |
| Wong 2023 [19] | Self-management skills; Behavioral activation; Medication adherence | No (described narratively using COM-B) | COM-B model; Mechanisms of Action; BCT Taxonomy | Video-conference psychoeducation; individual behavioral coaching; SMS text messaging |
| Craven 2025 [20] | Return-to-work knowledge; Self-efficacy; Communication skills (survivor and employer) | No (described narratively) | Multiple BCTs (e.g., instruction, modeling, action planning) | Two self-guided eLearning packages (survivor and employer versions); interactive activities; downloadable PDF tools |

Abbreviations: BP, blood pressure; BCT, Behavior Change Technique; COM-B, Capability-Opportunity-Motivation-Behavior; HCP, healthcare professional; ICF, International Classification of Functioning; TDF, Theoretical Domains Framework.
