# Supplementary material for: A systematic review on using intervention mapping to guide behavioral interventions for improving stroke patient outcomes
Source: Front Med (Lausanne). 2026 Jun 29;13:1850511. doi: 10.3389/fmed.2026.1850511 (PMC13357211; doi:10.3389/fmed.2026.1850511)
Supplement: Supplementary file 2 [file Supplementary_file_1.DOCX]

**Supplementary File 1: Complete Search Strategies for All Databases**

**PubMed:**

#1 "Stroke"[Mesh] OR "Stroke, Lacunar"[Mesh] OR "Stroke Rehabilitation"[Mesh] OR stroke[tiab] OR poststroke[tiab] OR post-stroke[tiab] OR cerebrovascular accident*[tiab] OR CVA[tiab] OR brain vascular accident*[tiab] OR cerebral infarct*[tiab] OR cerebral ischemia[tiab] OR brain ischemia[tiab] OR hemiplegia[tiab] OR hemiparesis[tiab]

#2 "Intervention Mapping"[tiab] OR ("intervention" AND "mapping")[tiab] OR "IM protocol*"[tiab] OR "IM framework"[tiab] OR "IM approach"[tiab] OR "IM methodology"[tiab]

#3 "Behavior Therapy"[Mesh] OR "Health Behavior"[Mesh] OR "Patient Compliance"[Mesh] OR "Self-Management"[Mesh] OR behavioral intervention*[tiab] OR behaviour intervention*[tiab] OR health promotion[tiab] OR lifestyle intervention*[tiab] OR self-management[tiab] OR self care[tiab] OR rehabilitation adherence[tiab] OR treatment adherence[tiab] OR exercise therapy[tiab] OR cognitive training[tiab]

#4 #1 AND #2 AND #3

**EmBase:**

#1 cerebrovascular disease/ OR exp brain infarction/ OR exp cerebrovascular accident/ OR stroke/ OR stroke rehabilitation/ OR stroke patient/

#2 (stroke OR poststroke OR post-stroke OR cerebrovascular accident* OR CVA OR cerebral infarct* OR cerebral ischemia OR brain ischemia OR hemiplegia OR hemiparesis).ti,ab,kw.

#3 #1 OR #2

#4 intervention mapping.ti,ab,kw. OR (intervention NEAR/2 mapping).ti,ab,kw. OR "IM protocol*".ti,ab,kw. OR "IM framework".ti,ab,kw.

#5 behavior therapy/ OR health behavior/ OR patient compliance/ OR self care/ OR rehabilitation/ OR exp exercise/ OR lifestyle/

#6 (behavior* intervention* OR behaviour* intervention* OR health promotion OR lifestyle intervention* OR self-management OR self care OR rehabilitation adherence OR treatment adherence OR exercise therapy OR cognitive training).ti,ab,kw.

#7 #5 OR #6

#8 #3 AND #4 AND #7

**Cochrane Library:**

#1 [mh "Stroke"] or [mh "Stroke Rehabilitation"] or [mh "Cerebrovascular Disorders"]

#2 (stroke or poststroke or post-stroke or cerebrovascular accident* or CVA or cerebral infarct* or cerebral ischemia or brain ischemia or hemiplegia or hemiparesis):ti,ab,kw

#3 #1 or #2

#4 "Intervention Mapping":ti,ab,kw or (intervention near/2 mapping):ti,ab,kw or "IM protocol":ti,ab,kw or "IM framework":ti,ab,kw

#5 [mh "Behavior Therapy"] or [mh "Health Behavior"] or [mh "Patient Compliance"] or [mh "Self-Management"]

#6 (behavior* intervention* or behaviour* intervention* or health promotion or lifestyle intervention* or self-management or self care or rehabilitation adherence or treatment adherence or exercise therapy or cognitive training):ti,ab,kw

#7 #5 or #6

#8 #3 and #4 and #7

**Web of Science:**

#1 TS=(stroke OR poststroke OR "post-stroke" OR "cerebrovascular accident*" OR CVA OR "cerebral infarct*" OR "cerebral ischemia" OR "brain ischemia" OR hemiplegia OR hemiparesis)

#2 TS=("Intervention Mapping" OR "intervention mapping" OR "IM protocol*" OR "IM framework" OR "IM approach")

#3 TS=("behavior* intervention*" OR "behaviour* intervention*" OR "health promotion" OR "lifestyle intervention*" OR self-management OR "self care" OR "rehabilitation adherence" OR "treatment adherence" OR "exercise therapy" OR "cognitive training")

#4 #3 AND #2 AND #1

**CINAHL:**

S1 (MH "Stroke") OR (MH "Stroke Patients") OR (MH "Stroke Rehabilitation") OR TI (stroke OR poststroke OR "post-stroke" OR cerebrovascular accident* OR CVA) OR AB (stroke OR poststroke OR "post-stroke" OR cerebrovascular accident* OR CVA)

S2 TI ("Intervention Mapping" OR "IM protocol*" OR "IM framework") OR AB ("Intervention Mapping" OR "IM protocol*" OR "IM framework")

S3 (MH "Behavior Therapy") OR (MH "Health Behavior") OR (MH "Patient Compliance") OR (MH "Self-Management") OR TI (behavior* intervention* OR behaviour* intervention* OR self-management OR "exercise therapy") OR AB (behavior* intervention* OR behaviour* intervention* OR self-management OR "exercise therapy")

S4 S1 AND S2 AND S3
